# Sequencing of mitochondrial genomes of nine *Aspergillus* and *Penicillium* species identifies mobile introns and accessory genes as main sources of genome size variability

**DOI:** 10.1186/1471-2164-13-698

**Published:** 2012-12-12

**Authors:** Vinita Joardar, Natalie F Abrams, Jessica Hostetler, Paul J Paukstelis, Suchitra Pakala, Suman B Pakala, Nikhat Zafar, Olukemi O Abolude, Gary Payne, Alex Andrianopoulos, David W Denning, William C Nierman

**Affiliations:** 1 The J. Craig Venter Institute, 9704 Medical Center Drive, , Rockville, MD, 20850, USA; 2 Department of Chemistry and Biochemistry, University of Maryland, College Park, MD, 20742, USA; 3 Institute for Genome Sciences, University of Maryland School of Medicine, Baltimore, MD, 21201, USA; 4 Department of Plant Pathology, North Carolina State University, Raleigh, NC, 27695, USA; 5 Department of Genetics, University of Melbourne, Victoria, 3010, Australia; 6 The University of Manchester and Manchester Academic Health Science Centre, Manchester, UK

## Abstract

**Background:**

The genera *Aspergillus* and *Penicillium* include some of the most beneficial as well as the most harmful fungal species such as the penicillin-producer *Penicillium chrysogenum* and the human pathogen *Aspergillus fumigatus*, respectively. Their mitochondrial genomic sequences may hold vital clues into the mechanisms of their evolution, population genetics, and biology, yet only a handful of these genomes have been fully sequenced and annotated.

**Results:**

Here we report the complete sequence and annotation of the mitochondrial genomes of six *Aspergillus* and three *Penicillium* species: *A*. *fumigatus*, *A*. *clavatus*, *A*. *oryzae*, *A*. *flavus*, *Neosartorya fischeri* (*A*. *fischerianus*), *A*. *terreus*, *P*. *chrysogenum*, *P*. *marneffei*, and *Talaromyces stipitatus* (*P*. *stipitatum*). The accompanying comparative analysis of these and related publicly available mitochondrial genomes reveals wide variation in size (25–36 Kb) among these closely related fungi. The sources of genome expansion include group I introns and accessory genes encoding putative homing endonucleases, DNA and RNA polymerases (presumed to be of plasmid origin) and hypothetical proteins. The two smallest sequenced genomes (*A*. *terreus* and *P*. *chrysogenum*) do not contain introns in protein-coding genes, whereas the largest genome (*T*. *stipitatus*), contains a total of eleven introns. All of the sequenced genomes have a group I intron in the large ribosomal subunit RNA gene, suggesting that this intron is fixed in these species. Subsequent analysis of several *A*. *fumigatus* strains showed low intraspecies variation. This study also includes a phylogenetic analysis based on 14 concatenated core mitochondrial proteins. The phylogenetic tree has a different topology from published multilocus trees, highlighting the challenges still facing the *Aspergillus* systematics.

**Conclusions:**

The study expands the genomic resources available to fungal biologists by providing mitochondrial genomes with consistent annotations for future genetic, evolutionary and population studies. Despite the conservation of the core genes, the mitochondrial genomes of *Aspergillus* and *Penicillium* species examined here exhibit significant amount of interspecies variation. Most of this variation can be attributed to accessory genes and mobile introns, presumably acquired by horizontal gene transfer of mitochondrial plasmids and intron homing.

## Background

The genera *Aspergillus* and *Penicillium* contain some of the most beneficial as well as the most harmful fungal species. They include many industrial producers of important antibiotics, enzymes and pharmaceuticals, which have brought about a massive transformational impact on human health [1]. Several other species are extensively used in production of foods or other useful compounds. Pathogenic fungi, on the other hand, can significantly affect livestock and crops, both as agents of infection and by means of contamination with mycotoxins, and they commonly cause allergic and occasionally life-threatening infections in humans [2]. Fungal infections in humans are notoriously difficult to diagnose and treat, especially in immunocompromised patients. To achieve a better understanding of their biology, nuclear genomes of several *Aspergillus* and *Penicillium* species have been sequenced, yet only a handful of mitochondrial genomes have been sequenced and annotated.

With a few notable exceptions such as *Podospora anserina*[3], *Chaetomium thermophilum*[4], and *Rhizoctonia solani* (S. Pakala, unpublished), fungal mitochondrial genomes are small, with an average size of 44,681 bp (based on the fungal mitochondrial genomes in the NCBI organelle database, January 2012). For a fraction of the cost required to sequence a nuclear genome, mitochondrial genomic sequences may provide vital clues into the evolution, population genetics, and biology of these fungi. Aside from energy metabolism, mutations in mitochondrial genes have been linked to cellular differentiation, cell death and senescence pathways, as well as drug resistance and hypovirulence [5-9]. The widespread uniparental inheritance and high copy number of these organelles make them promising markers for cost-effective species identification and for studying fungal population structure [10]. Mitochondrial DNA can be a rich source of novel genotyping markers due to the presence of highly mobile introns in many fungal mitochondria [11]. Finally, fungal mitochondria may serve as valuable experimental models for studies of human heart and muscle diseases linked to mitochondrial dysfunction [12]. Mitochondrial biology is gaining notice with the advent of so called “three parent in vitro fertilization” as a means of producing disease free children from a mother with an inherited mitochondrial genetic disease [13]. In *Aspergillus fumigatus* the advent of the ability to perform sexual crosses will potentially allow for the genetic analysis of mitochondrial mutations and their phenotypes.

Despite their importance, only a few complete *Aspergillus* and *Penicillium* mitochondrial genomes have been reported [14-17]. In *A*. *fumigatus*, the ratio of mitochondrial to nuclear genomes is 12:1, based on optical mapping [18]. Although mitochondrial sequence reads are generated in every eukaryotic genome sequencing project, most studies only report nuclear genomes. As a result, little is known about the mitochondrial genome organization in many important fungi, such as the human pathogen *A*. *fumigatus* and the penicillin producer *P*. *chrysogenum*, which impedes their functional studies. Here we report the complete sequence and annotation of mitochondrial genomes of six *Aspergillus* and three *Penicillium* species. The accompanying comparative analysis of these and related publicly available genomes provides insight into mitochondrial genome organization, distribution of group I introns and plasmid-encoded genes, and phylogenetic relationships among these fungi.

## Results and discussion

### Assembly and annotation of mitochondrial genomes

The following mitochondrial genomic DNAs were sequenced, assembled, and/or annotated in this study: *Aspergillus fumigatus* AF293, *Aspergillus fumigatus* A1163, *Aspergillus fumigatus* 210, *Aspergillus clavatus* NRRL 1, *Aspergillus oryzae* RIB40, *Aspergillus flavus* NRRL 3357, *Neosartorya fischeri* NRRL 181 (teleomorph of *Aspergillus fischerianus*), *Aspergillus terreus* NIH 2624, *Penicillium chrysogenum* 54–1255, *Penicillium marneffei* ATCC 18224, and *Talaromyces stipitatus* ATCC 10500 (teleomorph of *Penicillium stipitatum*) (Table 1 and Additional file 1). Additional strains in the process of being sequenced were also analyzed with respect to SNPs and DIPs. Sequence reads for *A*. *fumigatus* AF293, *A*. *fumigatus* A1163, *A*. *clavatus*, *N*. *fischeri* and *P*. *chrysogenum* were generated in the course of nuclear genome sequencing projects [18-21]. The *A*. *oryzae* mitochondrial genome [16] was re-annotated in this study to include protein coding genes. The *A*. *terreus* mitochondrial genome was assembled using Sanger reads obtained from GenBank [GenBank:AAJN00000000]. After being trimmed and rotated, mitochondrial sequences were processed through the standard J. Craig Venter Institute (JCVI) annotation pipeline to ensure annotation consistency.

**Table 1:** ***Aspergillus *and *Penicillium *mitochondrial genome statistics**

**Genome**^**a**^	**length of mitochondrial genome (bp)**	**GC %**	**Protein-coding genes**	**Protein-coding genes with introns**	**Length (and number) of introns in protein-coding genes (bp)**	**Length of intron in large subunit rRNA (bp)**	**tRNAs**	**intronic ORFS**	**Length (and number) of non-intronic accessory genes (bp)**	**length of nuclear genome (bp)**
***A. terreus NIH 2624***	24,658	27.1	15	0	0 (0)	1,705	27	1	0 (0)	29,331,195
***P. chrysogenum 54-1255***	27,017	24.7	16	0	0 (0)	1,678	28	1	1,227 (1)	32,223,735
***A. oryzae RIB40***^**b**^	29,202	26.2	17	1	1,780 (1)	1,703	26	2	2,010 (2)	37,088,582
***A. flavus NRRL 3357***	29,205	26.2	17	1	1,776 (1)	1,703	27	2	2,004 (1)	36,892,344
***A. fumigatus A1163***	30,696	25.5	19	1	2,020 (1)	1,720	31	3	1,410 (2)	29,205,420
*A. niger N909*	31,103	26.9	16	2	1,502 (2)	1,800	25	0	1,674 (2)	not available
***A. fumigatus AF210***	31,762	25.4	20	1	2,020 (1)	1,720	31	3	2,253 (3)	not available
***A. fumigatus AF293***	31,765	25.4	20	1	2,020 (1)	1,720	31	3	2,253 (3)	29,384,958
*A. nidulans FGSC A4*	33,227	24.9	20	2	4,108 (5)	1,689	28	4	1,524 (2)	29,828,291
*A. tubingensis 0932*	33,656	26.8	16	2	3,690 (4)	1,794	25	0	1,647 (2)	not available
***N. fischeri NRRL 181***	34,373	25.4	22	1	3,429 (2)	1,721	28	4	2,559 (4)	31,770,017
***A. clavatus NRRL 1***	35,056	25.0	21	2	4,304 (3)	1,719	26	4	4,509 (3)	27,859,441
***P. marneffei ATCC 18224***	35,432	24.6	26	3	9,775 (9)	1,672	30	10	768 (2)	28,643,865
*P. marneffei MP1*	35,438	24.6	25	3	9,776 (9)	1,672	30	9	1,647 (2)	not available
***T. stipitatus ATCC 10500***	36,351	24.9	26	3	12,140 (11)	1,713	27	12	0 (0)	35,685,443

^a^Genomes annotated at JCVI are shown in bold.

^b^Protein coding genes were annotated at JCVI.

### Mitochondrial genome size variation and sources of genome expansion

**Figure 1:**
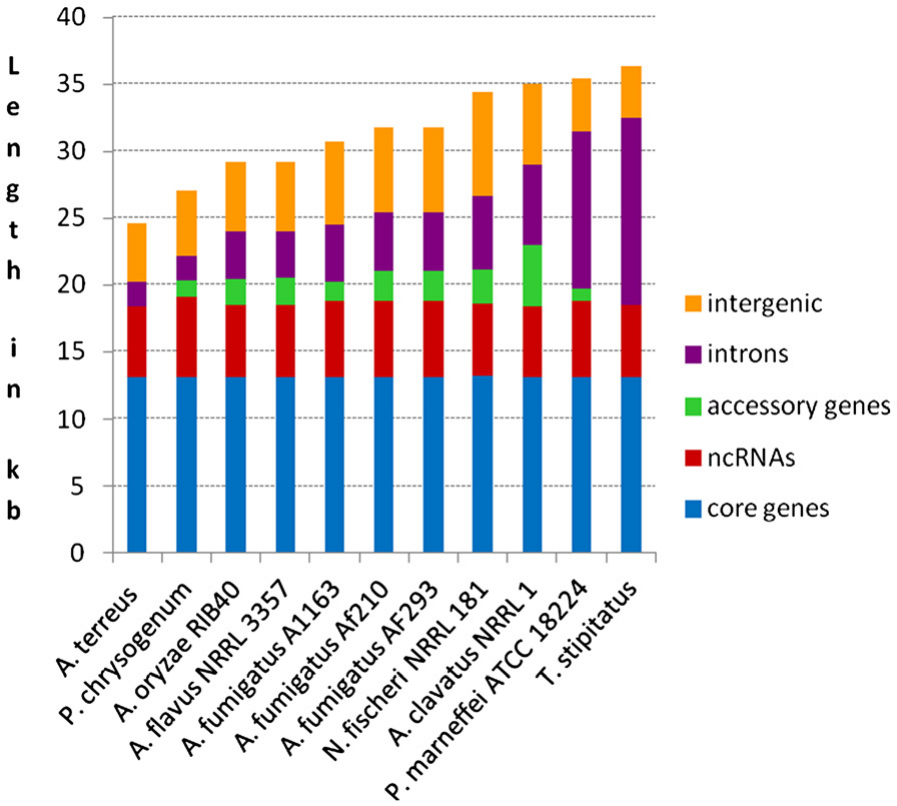
**Contributions from core and accessory genes, ncRNAs, intronic and intergenic regions, to mitochondrial genomes.** Each vertical bar represents the length of a mitochondrial genome.

The sequenced *Aspergillus* and *Penicillium* mitochondrial genomes showed remarkable variation in size, ranging from 24,658 bp to 36,351 bp (Table 1 and Figure 1). The differences in length can be primarily attributed to the number and length of the introns present in the genomes. For example, protein coding genes in the two smallest genomes, *A*. *terreus* and *P*. *chrysogenum*, do not contain introns, whereas the larger genomes contain one or more introns within ORFs. In contrast, the largest mitochondrial genome, *T*. *stipitatus*, contains a total of 11 introns. The presence and number of accessory genes also contribute to the larger size of some of the mitochondrial genomes. Repeat content within the analyzed species was determined to be insignificant (0 – 1% of the genomes, data not shown). Comparison of nuclear genome sizes shows no correlation with the mitochondrial DNA sizes (Table 1).

Comparative analysis of 11 *A*. *fumigatus* strains showed little intraspecies variation in their mitochondrial DNA (Additional file 2). To identify single nucleotide polymorphisms (SNPs) and deletion insertion polymorphisms (DIPs), Illumina reads of 10 *A*. *fumigatus* strains, generated in the course of a genome sequencing study (Nierman, unpublished), were aligned against the AF293 mitochondrial DNA. The analysis showed few SNPs and no significant DIPs. In total, we identified 15 candidate SNPs. The number of SNPs in individual strains varied from zero (F15861 and F15767) to nine (AF210). Six SNPs were located in intergenic regions, and nine were located in coding regions including eight non-synonymous SNPs. Five of these eight non-synonymous SNPs were found in *cob*, *cox1*, *nad1*, and *nad2* genes.

A 1.1 kb deletion was detected in strain A1163 (30,696 bp) compared to strains AF293 (31,765 bp) and AF210 (31,762 bp). The deletion in A1163 was confirmed by PCR using primers flanking the missing region. In AF293 and AF210, the region contains an 843-bp open reading frame (AFUA_m0390, AFUD_m0390; hypothetical protein), which is missing in A1163. A putative homolog of this hypothetical protein (NFIA_m0370) is present in the closely-related *N*. *fischeri* mitochondrial genome, suggesting a recent gene loss in *A*. *fumigatus* A1163.

Likewise, only minor differences were found between mitochondrial genomes of *A*. *oryzae* and *A*. *flavus* as well as between two strains of *P*. *marneffei* (ATCC 18224 and MP1). The *P*. *marneffei* genomes are 99.9% identical and their sizes differ only by 6 bp (Table 1). The genomes of *A*. *flavus* and *A*. *oryzae* differ in size by 3 bp and are also 99.9% identical. It should be noted that some of the strains analyzed were genetically modified during strain development. For example, the *P*. *chrysogenum* strain was developed as a result of an extensive strain improvement programs, which may have affected its mitochondrial DNA as well [20].

Our results are consistent with previous studies of mitochondrial intraspecies polymorphism. With a few exceptions, most Pezizomycotina mitochondrial genomes show little variation. By contrast, *Aspergillus japonicus*, *P*. *anserina*, *Neurospora crassa* and some other fungi exhibit significant mitochondrial intraspecies polymorphism and genome size variation, which has been attributed to mobile introns [11]. Thus, population surveys based on RFLP have demonstrated the presence of different mitochondrial haplotypes in wild-type subpopulations of *P*. *anserina*[22].

### Core mitochondrial genes

**Figure 2:**
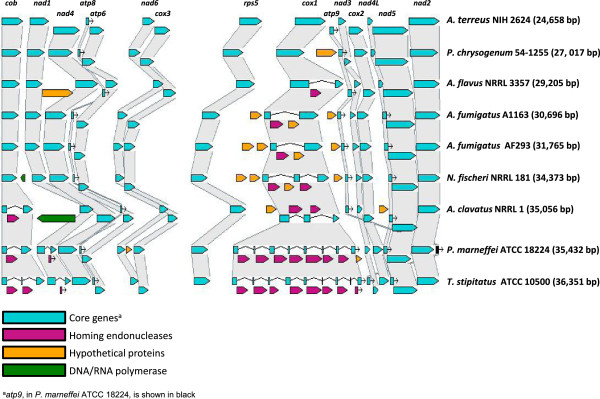
**Conservation of gene order in mitochondrial genomes.** A SynView representation of the protein-coding genes in the *Aspergillus* and *Penicillium* mitochondrial genomes annotated in this study. Clusters of orthologous core genes (blue) are connected by regions shaded in grey. Accessory genes are homing endonucleases (pink), hypothetical proteins (orange) and DNA/RNA polymerases (green). The synteny of the core genes is maintained with the exception of the location of *atp9* in *P. marneffei* 18224 (black).

All sequenced *Aspergillus* and *Penicillium* mitochondrial genomes contain 14 core genes involved in oxidative phosphorylation, ATP synthesis and mitochondrial protein synthesis, all present on the forward strand (Additional file 3). In addition, these genomes carry a complete set of tRNAs, the small and large subunits of ribosomal RNA, and the mitochondrial ribosomal protein S5. Additional file 4 depicts the protein-coding genes and non-coding RNAs in the reference mitochondrial genome of *A. fumigatus* AF293. The core genes share a high level of sequence conservation (Additional files 5 and 6) and synteny. Figure 2 shows the conservation of gene order of the core genes in all the *Aspergillus* and most of the *Penicillium* mitochondrial genomes annotated in this study. The exception is the *atp9* gene, which is located between *nad2* and *cob* in *P*. *marneffei* ATCC 18224). In all the other genomes, *atp9* lies between *cox1* and *nad3*. The number of tRNA genes varies from 25 to 31 with no particular correlation between the number and mitochondrial or nuclear genome size (Table 1).

**Figure 3:**
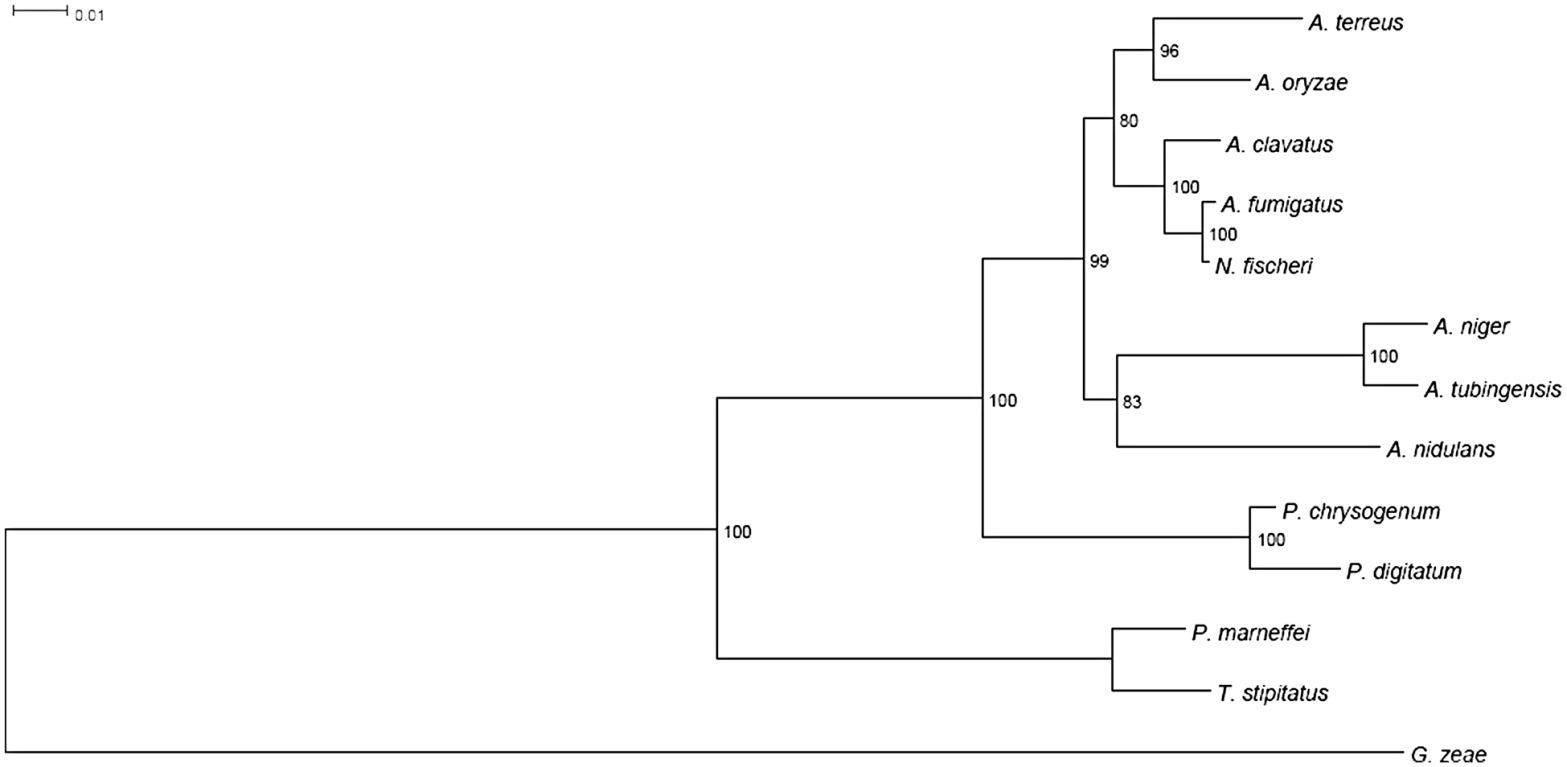
**Maximum Likelihood tree showing the phylogenetic relationships among the sequenced *Aspergillus *and *Penicillium *species.** The tree is based on 14 concatenated core mitochondrial proteins from 13 genomes. *Gibberella zeae* was used as an outgroup. Branch lengths correspond to substitutions per site calculated using a Maximum Likelihood approach. Identical topology was predicted using the Maximum Parsimony approach.

To explore the possibility that the core genes can provide new insights into *Aspergillus* evolution, we performed a phylogenetic analysis of 14 concatenated core proteins encoded by 13 *Aspergillus* and *Penicillium* mitochondrial genomes (Figure 3). The obtained phylogenetic tree clusters the *A*. *nidulans* and *A*. *niger* nodes together with a bootstrap support of 83%. Notably, *A*. *terreus* and *A*. *oryzae* form another group with bootstrap support of 80%. The tree topology also indicates that *P*. *chrysogenum* is not closely related to *P*. *marneffei* and *T*. *stipitatus*. This finding is consistent with morphological observations [20,23,24]. The topology of the Maximum Parsimony based tree is consistent with that of the Maximum-Likelihood tree (data not shown).

We have also examined individual mitochondrial genes (*cox1*, *cob*, *nad5*) and rDNA internal-transcribed spacers (ITS), which have been proposed as markers for species identification and classification to complement common nuclear DNA markers [10]. Phylogenetic analysis based on these individual protein coding and non-coding genes did not yield phylogenies with >75% bootstrap support for any of the trees (data not shown) and/or did not have enough power to resolve the relationships between the species. The single-gene trees show incompatible topologies with each other and with the topology obtained from the 14 concatenated proteins (Figure 3). This confirms that individual mitochondrial genes are not suitable for species identification in *Aspergillus* species. As shown previously [10], the presence of introns makes *cox1* and other mitochondrial genes poor candidates for building fungal phylogenies, although *cox1* is often used in animal phylogenetic studies.

In contrast, our results suggest that the concatenated core mitochondrial proteins can be of value for species level phylogeny construction. Interestingly, the concatenated tree topology is identical to the topology obtained previously from over 100 concatenated nuclear proteins with identical number of introns among orthologs [20]. The *A*. *terreus* and *A*. *oryzae* grouping is also supported by previously published studies based on multilocus phylogenetic analysis of both nuclear ribosomal and protein-coding genes [25] and concatenated nuclear protein-coding genes [26,27]. However, the *A*. *terreus* and *A*. *oryzae* nodes form two separate groups in phylogenies based on only nuclear ribosomal genes [28]. Our analysis highlights the challenges still facing the *Aspergillus* systematics including the unresolved *Aspergillus* tree backbone.

### Accessory mitochondrial genes

In addition to the core set, most mitochondrial genomes contain accessory genes. The most compact genome (*A*. *terreus*), contains only the core set of mitochondrial genes, while other genomes contain at least one accessory gene. Some accessory genes are shared by a subset of closely related mitochondrial genomes, but do not show sequence similarity to other genes in public databases. These genes have been annotated as ‘hypothetical’. Notably, *A*. *fumigatus* strains AF293 and A1163 contain 3 hypothetical genes each with > 99% identity.

In contrast, two accessory genes, *A*. *clavatus* ACLA_m0040 and *N*. *fischeri* NFIA_m0030, share similarity with mitochondrial DNA and RNA polymerase genes from distantly related fungi. They are also the only two protein-coding genes on the reverse strand, and are located between *cob* and *nad1* in their respective genomes. ACLA_m0040 has a potential frame shift and thus appears to be a pseudogene. Homologous DNA polymerase sequences are found within the main mitochondrial genome (as in *Glomerella graminicola* M1.001) or on linear mitochondrial plasmids (as in pAL2-1 of *P*. *anserina*, pClK1 of the phytopathogenic fungus *Claviceps purpurea* and pFP1 from *Fusarium proliferatum*). NFIA_m0030 is present as a C-terminal fragment (lacking a start codon) and is most similar to the RNA polymerase genes found on linear plasmids in *Blumeria graminis f*. *sp*. *hordei* (pBgh) and *C*. *purpurea* (pClK1).

The similarity to mitochondrial plasmid-encoded genes suggests that both *A*. *clavatus* and *N*. *fischeri* polymerase genes were acquired by horizontal gene transfer followed by integration of ancestral mitochondrial plasmids into mitochondrial DNA. Indeed, linear fungal mitochondrial plasmids typically encode DNA and RNA polymerases, while circular plasmids have a single gene for a DNA polymerase and a reverse transcriptase [29]. In several studies, plasmids integrated into mitochondrial DNA have been implicated in the mechanism underlying mycelial senescence in fungi [30-32].

Another set of accessory genes annotated in *Aspergillus* and *Penicillium* species encodes putative homing endonucleases. Although these genes (HEGs for “homing endonuclease genes”) can be found within introns and intergenic regions, in this study all HEGs were associated with “homing” group I introns (see next section). Based on sequence conservation, all HEGs were assigned to either LAGLIDADG or GIY-YIG families. The association between HEGs and introns is found in many other species. Homing introns are considered highly mobile, invasive genetic elements common in fungi and plants, but they can be also found in some animals and prokaryotes. They propagate via the double-strand-break-repair pathway into the specific target sequence (“homing site”), which is recognized and cleaved by the endonuclease [33]. Some endonucleases can also function as maturases by facilitating self-splicing of introns [34,35]. HEGs themselves are considered mobile and are typically bounded by two halves of the homing site (15–45 bp). HEGs are considered selfish elements, but may also contribute to mitochondrial DNA integrity [36]. The variability of HEGs in *Aspergillus* and *Penicillium* species can be exploited to develop phylogenetic markers that might be useful for differentiation of various species. Variants of HEGs are now being engineered for targeted cleavage of genomic sequences with potential applications in biotechnology, medicine and agriculture [37].

### Diversity, evolution and origin of mitochondrial group I intron insertions

Most fungal mitochondrial genomes sequenced to date contain one or more group I or group II introns. In the Pezizomycotina subphylum (to which all these species belong), the largest number of mitochondrial introns, a total of 33, have been documented for *P*. *anserina*[3], while *Mycosphaerella graminicola* is currently the only species identified that lacks mitochondrial introns entirely [38].

The *Aspergillus* and *Penicillium* species sequenced here also have variable intron distribution. Similar to previous observations the number and insertion sites of these introns vary, even between closely related species, suggesting cyclical intron gain and loss through horizontal transfer. Our analysis shows that the variation in intron number is the primary source of difference in genome size (Figure 1). Thus, the *T*. *stipitatus* genome contains eleven introns, while *A*. *terreus* and *P*. *chrysogenum* genomes both contain only a single intron.

The mitochondrial DNA sequences analyzed here contain group I introns distributed between three protein coding genes and the large subunit ribosomal RNA (LSU) (Table 1). As observed in a variety of mitochondrial genomes [10], the *cox1* gene contains the most variable number of introns. For the *Aspergillus* species, *A*. *nidulans* has three *cox1* introns, *A*. *clavatus* and *N*. *fischeri* each have two, and *A*. *fumigatus*, *A*. *flavus* and *A*. *oryzae* contain a single intron. *A*. *terreus* is the only *Aspergillus* species in this study that does not contain an intron in the *cox1* gene. The *cox1* intron insertions sites also vary between the *Aspergillus* species. The *cox1* intron insertion site in the *A*. *fumigatus* species is seen in the closely related *N*. *fischeri* genome and in the more distantly related *P*. *marneffei* and *T*. *stipitatus* genomes. The *cox1* insertion site in *A*. *flavus* and *A*. *oryzae* is also present in *A*. *clavatus*.

For the *Penicillium* species, *P*. *marneffei* and *T*. *stipitatus* contain seven and eight *cox1* introns, respectively, while *P*. *chrysogenum* does not contain any *cox1* introns. Between *P*. *marneffei* and *T*. *stipitatus*, six of the intron insertion sites are identical. All of the *cox1* intron insertions sites have been previously observed in other distantly related fungi such as *Saccharomyces cerevisiae*, *P*. *anserina* and *N*. *crassa*[3,39,40].

The other two protein coding genes that contain group I introns are the *cob* and *nad1* genes. *A*. *nidulans* and *A*. *clavatus* contain a common *cob* intron, while *P*. *marneffei* contains a single intron and *T*. *stipitatu*s contains two *cob* introns. These two *Penicillium* species are also the only two in this study with an intron in the *nad1* gene. The *cox1*, *cob*, and *nad1* introns in all the species encode either a LAGLIDADG or GIY-YIG homing endonuclease (as discussed above).

**Figure 4:**
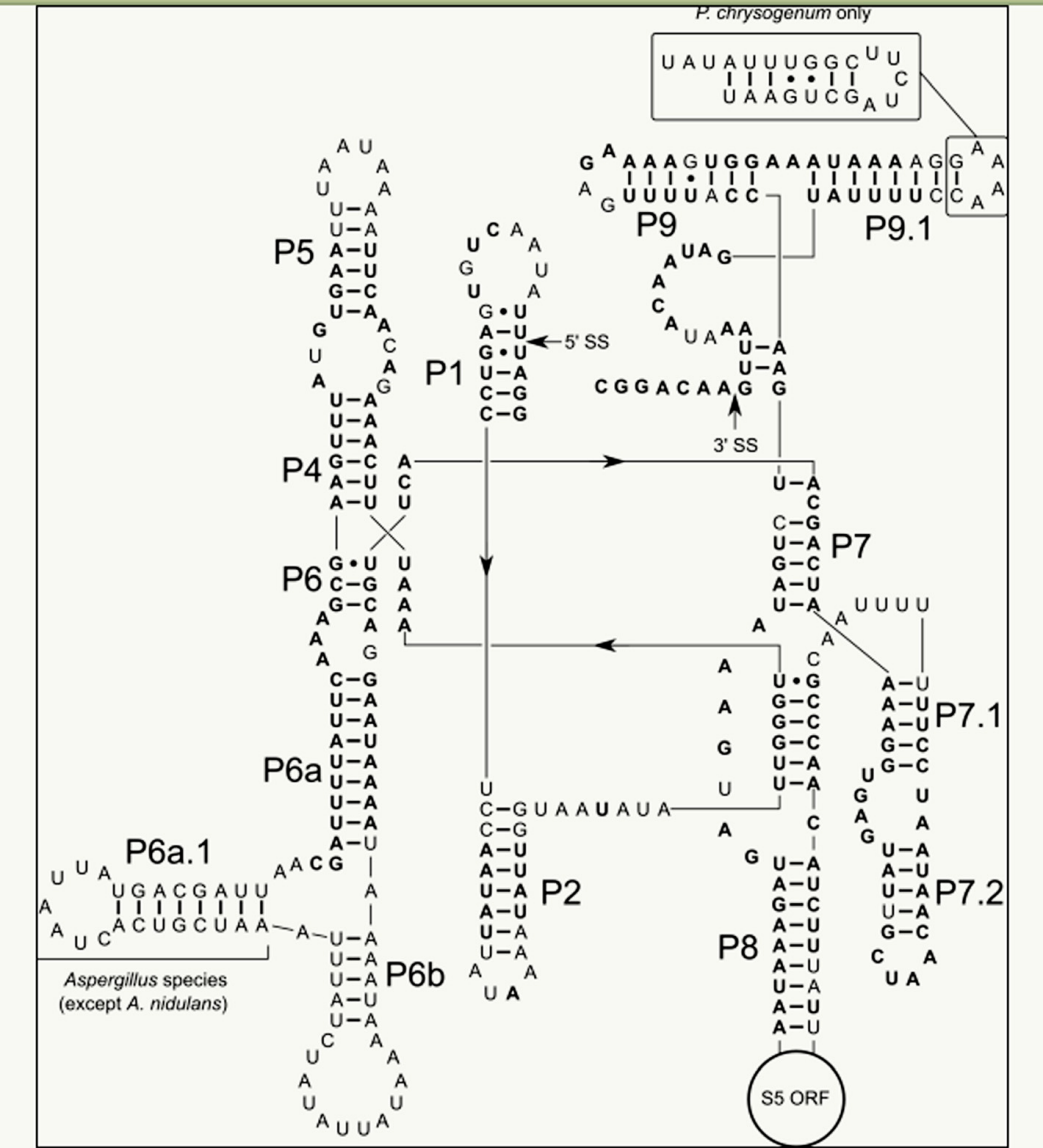
**Secondary structure of the mitochondrial LSU rRNA intron.** This intron is present in all the mitochondrial genomes described here, with the sequence shown being from *A. fumigatus*. Grey highlighted residues are identical in all species. All of the introns contain a mitochondrial S5 protein ORF in the P8 stem-loop. The primary difference between the *Aspergillus* and *Penicillium* species is the presence of an additional stem-loop structure, P6a.1, that is present in the *Aspergillus* species (except *A. nidulans*), but absent in the *Penicillium* species. *P. chrysogenum* is the only species to contain an extended P9.1 region. 5’SS: 5’ splice site; 3’SS: 3’ splice site.

By contrast, the one intron common to all the mitochondrial genomes described here is found at a single location in the LSU rRNA gene. These introns are closely related in secondary structure (Figure 4) with the only major difference between the genera being an additional stem-loop structure (P6c) present in all the *Aspergillus* introns (except *A*. *nidulans*), but absent in the *Penicillium* species. All Pezizomycotina mitochondria sequenced to date, with the exception of the Diothideomycetes (*Phaeosphaeria nodorum* and *Mycosphaerella graminicola*), contain a mitochondrial LSU rRNA intron inserted at this position. Intron insertions are also commonly found in the mitochondrial LSU gene in plants and other fungi.

Our results are consistent with two previously observed characteristics of Pezizomycotina mitochondrial LSU introns. First, these introns do not contain endonuclease ORFs, but do contain the mitochondrial ribosomal protein S5 ORF within the P8 stem-loop of the intron. In *S*. *cerevisiae* and other fungi, mitochondrial S5 is nuclear encoded and transported into the mitochondrial matrix [41] but is not encoded in the nuclear genome of any Pezizomycotina fungi sequenced to date. No studies have thoroughly examined the function of the mitochondrial S5 ORF, but decreased expression of mitochondrial S5 from the intron ORF in *N*. *crassa* leads to mitochondrial small ribosomal subunit assembly defects and decreased mitochondrial protein expression [42,43]. Second, though group I introns are normally considered self-splicing, the Pezizomycotina mitochondrial LSU introns tested to date cannot self-splice [44-47]. These introns require a mitochondrial tyrosyl-tRNA synthetase (TyrRS) as a structure-stabilizing splicing cofactor found only in Pezizomycotina [47].

The intron distribution in the genera described here suggests two distinct mechanisms of intron evolution within the lineage. The presence of homing endonucleases in all of the *cox1*, *cob1*, and *nad1* introns suggests they likely follow the previously proposed “omega” cycle of intron gain and loss [48]. In this model, horizontal transmission into an intronless site is promoted by a functional homing endonuclease, followed by endonuclease degradation, and eventually, intron loss. The cycle can then be restarted by a new horizontal transmission event. The sporadic distribution of introns in the protein coding genes analyzed here indicates that horizontal gene transfer may be quite common in the *Aspergillus* and *Penicillium* mitochondrial genomes. It also highlights the challenges associated with using *cox1* and other mitochondrial genes to build species phylogenies.

The mitochondrial LSU intron likely follows a slightly different evolutionary trajectory. The widespread distribution of mitochondrial LSU introns in Pezizomycotina that contain the S5 ORF suggests that this intron insertion event occurred after the divergence from the yeasts, and became fixed within the lineages. Fixation was likely due to the selective advantage from the S5 gene, but also from accumulated mutations in the intron, which resulted in its dependence on the nuclear encoded mitochondrial TyrRS splicing factor. This idea is supported by the observation that the Dithideomycetes, the only Pezizomycotina group that lacks mitochondrial LSU introns, contain degraded adaptations of the mitochondrial TyrRS splicing factor necessary for intron splicing [46]. A plausible scenario is that this degeneration occurred after mitochondrial LSU intron loss. It remains unclear how the Dithideomycetes have compensated for the loss of the S5 protein [38,49].

## Conclusions

We report here the complete sequence and annotation of mitochondrial genomes of six *Aspergillus* and three *Penicillium* species, which represent the two most significant genera among filamentous fungi. The study expands the genomic resources available to fungal biologists by providing mitochondrial genomes with consistent annotations. The accompanying comparative analysis of these and related publicly available genomes provides insight into genome organization and phylogenetic relationships among these organisms. By clustering together *A*. *terreus* and *A*. *oryzae*, the phylogenetic tree based on 14 concatenated core mitochondrial proteins has a different topology from some previously published single protein trees, but is similar to trees built using multiple nuclear proteins. This suggests that core genes in mitochondrial and nuclear genomes co-evolved in the *Aspergillus* lineage.

Despite the conservation of the core genes, mitochondrial genomes of *Aspergillus* and *Penicillium* species exhibit significant amount of interspecies variation consistent with experimental evidence for intraspecies horizontal transfer and recombination in mitochondrial DNA [50]. Most of this variation can be attributed to accessory genes and mobile introns, presumably acquired via swapping and integration of mitochondrial plasmid and intron homing followed by gene or intron loss. Annotated core and accessory genes can serve as complementary markers in future population genetics and evolution studies.

## Methods

### Sequencing and assembly

The following genomes were sequenced at JCVI using the Sanger technology: *A*. *fumigatus* AF293, *A*. *fumigatus* A1163, *A*. *clavatus* NRRL 1, *A*. *flavus* NRRL 3357, *N*. *fischeri* NRRL 181, *P*. *chrysogenum* 54–1255, *P*. *marneffei* ATCC 18224, and *T*. *stipitatus* ATCC 10500 (Additional file 1). The *A*. *fumigatus* AF210, genome was sequenced using a combination of 454 GS FLX Titanium instrument (Roche) and Illumina Genome Analyzer II (Illumina). The reads were assembled at JCVI using the Celera Assembler [51]. The *P*. *chrysogenum* mitochondrial genome was previously reported [20], but the sequence was not trimmed. We therefore trimmed, rotated, and re-annotated the original sequence to ensure annotation consistency. The *A*. *terreus* NIH 2624 genome was assembled at JCVI using Sanger reads or traces obtained from NCBI [GenBank:AAJN00000000], which were deposited by the Broad Institute. The *A*. *nidulans* FGSC A4 genome was assembled at JCVI using reads provided by the Broad Institute (with additional sequencing performed at JCVI). The remaining complete mitochondrial genome sequences used in this study were either obtained from NCBI (http://www.ncbi.nlm.nih.gov) or sequenced and assembled at JCVI (see Genome closure section below). The following mitochondrial genome sequences were obtained from NCBI: *A*. *niger* N909 [Genbank:DQ207726], *A*. *tubingensis* 0932 [Genbank:DQ217399], *A*. *oryzae* RIB 40 [Genbank:AP007176], *P*. *marneffei* MP1 [GenBank:AY347307] and *A*. *nidulans* FGSC A4 [GenBank:JQ435097].

### Genome closure

Mitochondrial contigs were completed using available *de novo* contigs and/or mapping to mitochondrial reference genomes. Seed contigs from *de novo* assemblies of all genomic data were analyzed for high coverage and for the presence of common mitochondrial genes. Simultaneously, sequence reads were mapped to known references to generate a read set for *de novo* assembly. Resulting mitochondrial contigs were iteratively extended through recruiting sequence data by aligning to contig edges until no gaps remained. All contigs were manually examined for quality. All contain at least 2 fold high quality coverage of every base and had evidence of circularity based on mate pairing and/or overlapping contig edges. The resulting scaffolds were trimmed and rotated to facilitate comparative analysis. The deletion in *A*. *fumigatus* A1163 was confirmed by PCR using primers flanking the missing region (Primers: AF1163C16842: 5^′^-ATTGTTCATTATTCTACAGTTAAGCC-3^′^ and AF1163C17705: 5^′^-AATTAGTATCCTCATCTTCCTTAGG-3^′^). Annotated scaffolds have been deposited in NCBI [GenBank:JQ346807, GenBank:JQ346808, GenBank:JQ346809, GenBank:JQ354994, GenBank:JQ354995, GenBank:JQ354996, GenBank:JQ354997, GenBank:JQ354998, GenBank:JQ354999, GenBank:JQ355000 and GenBank:JQ355001].

### SNP and DIP identification in *A*. *fumigatus* mitochondrial DNA

SNPs and small DIPs were predicted using CLC Genomics Workbench from CLC Bio (http://www.clc-bio.com). Illumina reads from target strains were mapped to the reference *A*. *fumigatus* AF293 mitochondrial genome. The mapping parameters used were 0.9 for Length fraction and 0.9 for Similarity. Non specific matches were ignored. Default cost parameters were used. To call SNPs and small DIPs, we used stringent parameters that were obtained from extensive manual evaluation of alignments. The following cut-offs were used in CLC to call SNPs: (i) read coverage is equal to or above 10; and (ii) variants supported by at least 99% of the reads. The quality of read alignments and the regions surrounding the called SNP locations were manually inspected. Low complexity regions were ignored. The SNPs that passed these filtering criteria were retained. MUMmer [52] and BLASTn [53] were used to check for the presence of large scale rearrangements and insertions/deletions.

### Repetitive regions identification

RepeatMasker (http://www.repeatmasker.org/) was used to check for the presence of high copy interspersed repeats and low complexity DNA sequences. PrintRepeats [54] (http://www.genome.ou.edu/miropeats.html) was used to identify low copy repeats.

### Mitochondrial genome annotation

All mitochondrial genomes analyzed in this study were annotated at JCVI, except for *A*. *niger*, *A*. *tubingensis*, *A*. *nidulans* and *P*. *marneffei* MP1 (Additional file 1). Mitochondrial *A*. *oryzae* tRNA genes were obtained from NCBI. Open reading frames (ORFs) were identified using Artemis [55] with genetic code 4. Functional assignments were made based on sequence similarity to characterized fungal mitochondrial proteins using BLASTp searches against NCBI databases. ORFs containing more than 100 amino acids, and no sequence homology to known genes, were designated as hypothetical genes. tRNA genes were identified using tRNAscan-SE and ribosomal RNA genes were identified using BLASTn [53]. Core mitochondrial-encoded genes were identified by all-against-all comparison using BLASTp.

### Group I intron annotation

Approximate group I intron insertion boundaries were initially established as interruptions in the protein-coding genes and LSU rRNA gene identified through BLASTp and BLASTn searches. Precise 5^′^ intron boundaries were determined by identifying the conserved U-G or C-G within the intron’s P1 stem. The 3^′^ intron boundaries were determined by identifying the G at the end of the intron and the ability for the downstream sequence to form the P10 guide sequence stem [47,56]. The identified boundaries were confirmed by tBLAST searches of the putative spliced products. The mitochondrial LSU group I intron secondary structures were constructed from the previously described *A*. *nidulans* secondary structure [47].

### Synteny analysis of core genes

OrthoMCL [57] was used to identify the orthologous relationships between the 15 core protein-coding genes. The first step was an all-against-all BLASTp search with an expect value of 1e-05. This was followed by the MCL clustering algorithm using default parameters and the main inflation value (−I) set to 1.5. The orthologous clusters were displayed in Figure 2 using SynView [58].

### Phylogenetic analysis

To generate phylogenetic trees, 14 core proteins encoded by 13 genomes were first concatenated and then aligned using Muscle [59]. Regions with poor alignments were removed with Gblocks using default settings [60]. Maximum-Likelihood (ML) trees were generated using the Randomized Axelerated Maximum Likelihood (RAxML) program [61]. Multiple ML trees were generated and the best-scoring tree was identified. 100 boot-strapped trees were generated and used to assign the boot strap support values to the best-scoring ML tree. The JTT amino acid substitution model was used with the Gamma model of rate heterogeneity. *Gibberella zeae* (anamorph *Fusarium graminearum*) was used as an outgroup. A Maximum Parsimony based tree was generated using the Protpars program of the PHYLIP package [62].

## Competing interests

The authors declare that they have no competing interests.

## Authors’ contributions

VJ and NFA performed the comparative analysis and interpretation of data and drafted the manuscript. VJ and OOA annotated the mitochondrial genomes. JH coordinated genome assembly and closure. PJP conducted intron annotation and analysis. SP, SBP and NZ performed phylogenetic, repeat content, comparative genomics, and other analyses. GP, AA, DWD and WCN made substantial contributions to the study conception and design and to the preparation of the manuscript. All authors have read and approved the final manuscript.

## Supplementary Material

Additional file 1 Sequence data sources. Click here for file

Additional file 2 **SNPs predicted in *A.fumigatus *strains with respect to the AF293 reference mitochondrial DNA.** Click here for file

Additional file 3 **Core and accessory protein-coding genes in *Aspergillus *and *Penicillium *mitochondrial genomes.** Click here for file

Additional file 4 **The *A.fumigatus *AF293 mitochondrial genome showing the protein-coding genes, and the non-coding RNAs.** Click here for file

Additional file 5 Protein sequences of the core mitochondrial genes. Click here for file

Additional file 6 Multiple sequence alignment of the core mitochondrial proteins. Click here for file

## References

[B1] Hoffmeister D Keller NP Natural products of filamentous fungi: enzymes, genes, and their regulation Nat Prod Rep 2007 24 2 393 416 17390002 10.1039/b603084j

[B2] Enoch DA Ludlam HA Brown NM Invasive fungal infections: a review of epidemiology and management options J Med Microbiol 2006 55 Pt 7 809 818 16772406 10.1099/jmm.0.46548-0

[B3] Cummings DJ McNally KL Domenico JM Matsuura ET The complete DNA sequence of the mitochondrial genome of Podospora anserina Curr Genet 1990 17 5 375 402 2357736 10.1007/BF00334517

[B4] Amlacher S Sarges P Flemming D van Noort V Kunze R Devos DP Arumugam M Bork P Hurt E Insight into structure and assembly of the nuclear pore complex by utilizing the genome of a eukaryotic thermophile Cell 2011 146 2 277 289 21784248 10.1016/j.cell.2011.06.039

[B5] Osiewacz HD Brust D Hamann A Kunstmann B Luce K Muller-Ohldach M Scheckhuber CQ Servos J Strobel I Mitochondrial pathways governing stress resistance, life, and death in the fungal aging model Podospora anserina Ann N Y Acad Sci 2010 1197 54 20536834 10.1111/j.1749-6632.2010.05190.x

[B6] Sanglard D Ischer F Bille J Role of ATP-binding-cassette transporter genes in high-frequency acquisition of resistance to azole antifungals in Candida glabrata Antimicrob Agents Chemother 2001 45 4 1174 1183 11257032 10.1128/AAC.45.4.1174-1183.2001 PMC90441

[B7] Ferrari S Sanguinetti M Torelli R Posteraro B Sanglard D Contribution of CgPDR1-regulated genes in enhanced virulence of azole-resistant Candida glabrata PLoS One 2011 6 3 e17589 21408004 10.1371/journal.pone.0017589 PMC3052359

[B8] Martins VP Dinamarco TM Soriani FM Tudella VG Oliveira SC Goldman GH Curti C Uyemura SA Involvement of an alternative oxidase in oxidative stress and mycelium-to-yeast differentiation in Paracoccidioides brasiliensis Eukaryot Cell 2011 10 2 237 248 21183691 10.1128/EC.00194-10 PMC3067407

[B9] Scheckhuber CQ Hamann A Brust D Osiewacz HD Cellular homeostasis in fungi: impact on the aging process Sub-cellular biochemistry 57 233 250 22094425 10.1007/978-94-007-2561-4_11

[B10] Santamaria M Vicario S Pappada G Scioscia G Scazzocchio C Saccone C Towards barcode markers in fungi: an intron map of Ascomycota mitochondria BMC Bioinforma 2009 10 Suppl 6 S15 10.1186/1471-2105-10-S6-S15 PMC2697638 19534740

[B11] Hamari Z Juhasz A Kevei F Role of mobile introns in mitochondrial genome diversity of fungi (a mini review) Acta Microbiol Immunol Hung 2002 49 2–3 331 335 12109166 10.1556/AMicr.49.2002.2-3.22

[B12] Wallace DC Mitochondrial DNA mutations in disease and aging Environ Mol Mutagen 2010 51 5 440 450 20544884 10.1002/em.20586

[B13] Tavare A Scientists are to investigate “three parent IVF” for preventing mitochondrial diseases BMJ (Clinical research ed) 2012 344 e540 10.1136/bmj.e540 22267657

[B14] Juhasz A Pfeiffer I Keszthelyi A Kucsera J Vagvolgyi C Hamari Z Comparative analysis of the complete mitochondrial genomes of Aspergillus niger mtDNA type 1a and Aspergillus tubingensis mtDNA type 2b FEMS Microbiol Lett 2008 281 1 51 57 18318841 10.1111/j.1574-6968.2008.01077.x

[B15] Juhasz A Engi H Pfeiffer I Kucsera J Vagvolgyi C Hamari Z Interpretation of mtDNA RFLP variability among Aspergillus tubingensis isolates Antonie Van Leeuwenhoek 2007 91 3 209 216 17043909 10.1007/s10482-006-9110-x

[B16] Machida M Asai K Sano M Tanaka T Kumagai T Terai G Kusumoto K Arima T Akita O Kashiwagi Y Genome sequencing and analysis of Aspergillus oryzae Nature 2005 438 7071 1157 1161 16372010 10.1038/nature04300

[B17] Woo PC Zhen H Cai JJ Yu J Lau SK Wang J Teng JL Wong SS Tse RH Chen R The mitochondrial genome of the thermal dimorphic fungus Penicillium marneffei is more closely related to those of molds than yeasts FEBS Lett 2003 555 3 469 477 14675758 10.1016/s0014-5793(03)01307-3

[B18] Nierman WC Pain A Anderson MJ Wortman JR Kim HS Arroyo J Berriman M Abe K Archer DB Bermejo C Genomic sequence of the pathogenic and allergenic filamentous fungus Aspergillus fumigatus Nature 2005 438 7071 1151 1156 16372009 10.1038/nature04332

[B19] Galagan JE Calvo SE Cuomo C Ma LJ Wortman JR Batzoglou S Lee SI Basturkmen M Spevak CC Clutterbuck J Sequencing of Aspergillus nidulans and comparative analysis with A. fumigatus and A. oryzae Nature 2005 438 7071 1105 1115 16372000 10.1038/nature04341

[B20] van den Berg MA Albang R Albermann K Badger JH Daran JM Driessen AJ Garcia-Estrada C Fedorova ND Harris DM Heijne WH Genome sequencing and analysis of the filamentous fungus Penicillium chrysogenum Nat Biotechnol 2008 26 10 1161 1168 18820685 10.1038/nbt.1498

[B21] Fedorova ND Khaldi N Joardar VS Maiti R Amedeo P Anderson MJ Crabtree J Silva JC Badger JH Albarraq A Genomic islands in the pathogenic filamentous fungus Aspergillus fumigatus PLoS Genet 2008 4 4 e1000046 18404212 10.1371/journal.pgen.1000046 PMC2289846

[B22] van Diepeningen AD Goedbloed DJ Slakhorst SM Koopmanschap AB Maas MF Hoekstra RF Debets AJ Mitochondrial recombination increases with age in Podospora anserina Mech Ageing Dev 2010 131 5 315 20226205 10.1016/j.mad.2010.03.001

[B23] Samson RA Pitt JI Advances in Penicillium and Aspergillus systematics 1985 New York: Plenum Press

[B24] Berbee M Yoshimura A Sugiyama J Taylor JW Is Penicillium monophyletic? An evaluation of phylogeny in the family Trichocomaceae from 18S, 5.8S And ITS ribosomal DNA sequence data Mycologia 1995 87 2 210 222

[B25] Geiser DM Samson RA Varga J Rokas A Witiak SM Varga J, Sampson RA A review of molecular phylogenetics in Aspergillus, and prospects for a robust genus-wide phylogeny Aspergillus in the genomics era 2008 Wageningen: Wageningen Academic Publishers 17 32

[B26] Pel HJ de Winde JH Archer DB Dyer PS Hofmann G Schaap PJ Turner G de Vries RP Albang R Albermann K Genome sequencing and analysis of the versatile cell factory Aspergillus niger CBS 513.88 Nature biotechnology 2007 25 2 221 231 10.1038/nbt1282 17259976

[B27] Rokas A Galagan JE Osmani SA, Goldman GH Aspergillus nidulans genome and a comparative analysis of genome evolution in Aspergillus The aspergilli: genomics, medical aspects, biotechnology, and research methods 2007 Boca Raton: CRC Press 43 55

[B28] Peterson SW Phylogenetic analysis of Aspergillus species using DNA sequences from four loci Mycologia 2008 100 2 205 226 18595197 10.3852/mycologia.100.2.205

[B29] Griffiths AJ Natural plasmids of filamentous fungi Microbiol Rev 1995 59 4 673 685 8531891 10.1128/mr.59.4.673-685.1995 PMC239394

[B30] Maheshwari R Navaraj A Senescence in fungi: the view from Neurospora FEMS Microbiol Lett 2008 280 2 135 143 18093134 10.1111/j.1574-6968.2007.01027.x

[B31] Maas MF Sellem CH Hoekstra RF Debets AJ Sainsard-Chanet A Integration of a pAL2-1 homologous mitochondrial plasmid associated with life span extension in Podospora anserina Fungal Genet Biol 2007 44 7 659 671 17166751 10.1016/j.fgb.2006.10.007

[B32] Court DA Griffiths AJ Kraus SR Russell PJ Bertrand H A new senescence-inducing mitochondrial linear plasmid in field-isolated Neurospora crassa strains from India Curr Genet 1991 19 2 129 137 1648454 10.1007/BF00326294

[B33] Belfort M Roberts RJ Homing endonucleases: keeping the house in order Nucleic Acids Res 1997 25 17 3379 3388 9254693 10.1093/nar/25.17.3379 PMC146926

[B34] Delahodde A Goguel V Becam AM Creusot F Perea J Banroques J Jacq C Site-specific DNA endonuclease and RNA maturase activities of two homologous intron-encoded proteins from yeast mitochondria Cell 1989 56 3 431 441 2536593 10.1016/0092-8674(89)90246-8

[B35] Wenzlau JM Saldanha RJ Butow RA Perlman PS A latent intron-encoded maturase is also an endonuclease needed for intron mobility Cell 1989 56 3 421 430 2536592 10.1016/0092-8674(89)90245-6

[B36] Basse CW Mitochondrial inheritance in fungi Curr Opin Microbiol 2010 13 6 712 719 20884279 10.1016/j.mib.2010.09.003

[B37] Stoddard BL Homing endonucleases: from microbial genetic invaders to reagents for targeted DNA modification Structure 2011 19 1 7 15 21220111 10.1016/j.str.2010.12.003 PMC3038549

[B38] Torriani SF Goodwin SB Kema GH Pangilinan JL McDonald BA Intraspecific comparison and annotation of two complete mitochondrial genome sequences from the plant pathogenic fungus Mycosphaerella graminicola Fungal Genet Biol 2008 45 5 628 637 18226935 10.1016/j.fgb.2007.12.005

[B39] Foury F Roganti T Lecrenier N Purnelle B The complete sequence of the mitochondrial genome of Saccharomyces cerevisiae FEBS Lett 1998 440 3 325 331 9872396 10.1016/s0014-5793(98)01467-7

[B40] Field DJ Sommerfield A Saville BJ Collins RA A group II intron in the Neurospora mitochondrial coI gene: nucleotide sequence and implications for splicing and molecular evolution Nucleic Acids Res 1989 17 22 9087 9099 2531370 10.1093/nar/17.22.9087 PMC335116

[B41] Goffeau A Barrell BG Bussey H Davis RW Dujon B Feldmann H Galibert F Hoheisel JD Jacq C Johnston M Life with 6000 genes Science New York, NY 996 274 5287 546 563–547 10.1126/science.274.5287.546 8849441

[B42] LaPolla RJ Lambowitz AM Mitochondrial ribosome assembly in Neurospora crassa. Purification of the mitochondrially synthesized ribosomal protein, S-5 J Biol Chem 1981 256 13 7064 7067 6453873

[B43] Lapolla RJ Lambowitz AM Mitochondrial ribosome assembly in Neurospora. Structural analysis of mature and partially assembled ribosomal subunits by equilibrium centrifugation in CsCl gradients J Cell Biol 1982 95 1 267 277 6216256 10.1083/jcb.95.1.267 PMC2112348

[B44] Guo QB Akins RA Garriga G Lambowitz AM Structural analysis of the Neurospora mitochondrial large rRNA intron and construction of a mini-intron that shows protein-dependent splicing J Biol Chem 1991 266 3 1809 1819 1824845

[B45] Kamper U Kuck U Cherniack AD Lambowitz AM The mitochondrial tyrosyl-tRNA synthetase of Podospora anserina is a bifunctional enzyme active in protein synthesis and RNA splicing Mol Cell Biol 1992 12 2 499 511 1531084 10.1128/mcb.12.2.499-511.1992 PMC364206

[B46] Hur M Geese WJ Waring RB Self-splicing activity of the mitochondrial group-I introns from Aspergillus nidulans and related introns from other species Curr Genet 1997 32 6 399 407 9388295 10.1007/s002940050294

[B47] Paukstelis PJ Lambowitz AM Identification and evolution of fungal mitochondrial tyrosyl-tRNA synthetases with group I intron splicing activity Proc Natl Acad Sci U S A 2008 105 16 6010 6015 18413600 10.1073/pnas.0801722105 PMC2329719

[B48] Goddard MR Burt A Recurrent invasion and extinction of a selfish gene Proc Natl Acad Sci U S A 1999 96 24 13880 13885 10570167 10.1073/pnas.96.24.13880 PMC24159

[B49] Hane JK Lowe RG Solomon PS Tan KC Schoch CL Spatafora JW Crous PW Kodira C Birren BW Galagan JE Dothideomycete plant interactions illuminated by genome sequencing and EST analysis of the wheat pathogen Stagonospora nodorum Plant Cell 2007 19 11 3347 3368 18024570 10.1105/tpc.107.052829 PMC2174895

[B50] Hamari Z Toth B Beer Z Gacser A Kucsera J Pfeiffer I Juhasz A Kevei F Interpretation of intraspecific variability in mtDNAs of Aspergillus niger strains and rearrangement of their mtDNAs following mitochondrial transmissions FEMS Microbiol Lett 2003 221 1 63 71 12694912 10.1016/S0378-1097(03)00165-4

[B51] Miller JR Delcher AL Koren S Venter E Walenz BP Brownley A Johnson J Li K Mobarry C Sutton G Aggressive assembly of pyrosequencing reads with mates Bioinformatics (Oxford, England) 2008 24 24 2818 2824 10.1093/bioinformatics/btn548 PMC2639302 18952627

[B52] Delcher AL Phillippy A Carlton J Salzberg SL Fast algorithms for large-scale genome alignment and comparison Nucleic Acids Res 2002 30 11 2478 2483 12034836 10.1093/nar/30.11.2478 PMC117189

[B53] Altschul SF Gish W Miller W Myers EW Lipman DJ Basic local alignment search tool J Mol Biol 1990 215 3 403 410 2231712 10.1016/S0022-2836(05)80360-2

[B54] Parsons JD Miropeats: graphical DNA sequence comparisons Comput Appl Biosci 1995 11 6 615 619 8808577 10.1093/bioinformatics/11.6.615

[B55] Rutherford K Parkhill J Crook J Horsnell T Rice P Rajandream MA Barrell B Artemis: sequence visualization and annotation Bioinformatics (Oxford, England) 2000 16 10 944 945 10.1093/bioinformatics/16.10.944 11120685

[B56] Michel F Westhof E Modelling of the three-dimensional architecture of group I catalytic introns based on comparative sequence analysis J Mol Biol 1990 216 3 585 610 2258934 10.1016/0022-2836(90)90386-Z

[B57] Li L Stoeckert CJ Jr Roos DS OrthoMCL: identification of ortholog groups for eukaryotic genomes Genome Res 2003 13 9 2178 2189 12952885 10.1101/gr.1224503 PMC403725

[B58] Wang H Su Y Mackey AJ Kraemer ET Kissinger JC SynView: a GBrowse-compatible approach to visualizing comparative genome data Bioinformatics (Oxford, England) 2006 22 18 2308 2309 10.1093/bioinformatics/btl389 16844709

[B59] Edgar RC MUSCLE: multiple sequence alignment with high accuracy and high throughput Nucleic Acids Res 2004 32 5 1792 1797 15034147 10.1093/nar/gkh340 PMC390337

[B60] Castresana J Selection of conserved blocks from multiple alignments for their use in phylogenetic analysis Mol Biol Evol 2000 17 4 540 552 10742046 10.1093/oxfordjournals.molbev.a026334

[B61] Stamatakis A Ludwig T Meier H RAxML-III: a fast program for maximum likelihood-based inference of large phylogenetic trees Bioinformatics (Oxford, England) 2005 21 4 456 463 10.1093/bioinformatics/bti191 15608047

[B62] Felsenstein J PHYLIP—phylogeny inference package (version 3.2) Cladistics 1989 5 164 166

