# Supplementary material for: Sequencing of mitochondrial genomes of nine Aspergillus and Penicillium species identifies mobile introns and accessory genes as main sources of genome size variability
Source: BMC Genomics. 2012 Dec 12;13:698. doi: 10.1186/1471-2164-13-698 (PMC3562157; doi:10.1186/1471-2164-13-698)

**Additional file 6**: The *A. fumigatus* AF293 mitochondrial genome showing the protein-coding genes, and the non-coding RNAs.


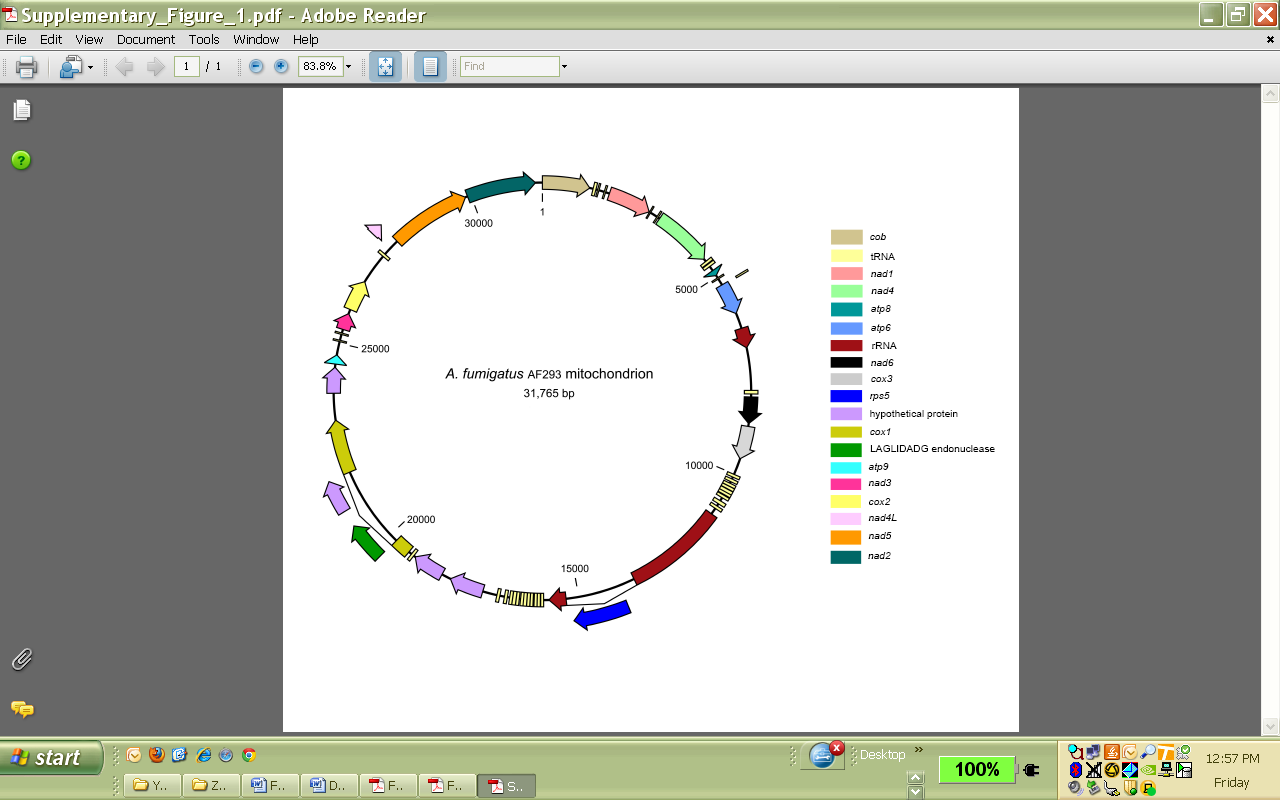

Supplement: Additional file 4 — The A.fumigatus AF293 mitochondrial genome showing the protein-coding genes, and the non-coding RNAs. [file 1471-2164-13-698-S4.doc]
